# ^p^NNS-Conjugated Chitosan Mediated IGF-1 and miR-140 Overexpression in Articular Chondrocytes Improves Cartilage Repair

**DOI:** 10.1155/2019/2761241

**Published:** 2019-03-21

**Authors:** Rong-lan Zhao, Xu-mei Zhang, Li-na Jia, Wei Song, Yan-li Sun, Xiang-ying Meng, Xiao-xiang Peng

**Affiliations:** ^1^Institute of Nanomedicine Technology, Department of Laboratory Medicine, Weifang Medical University, Weifang, Shandong 261053, China; ^2^Institutional Key Laboratory of Clinical Laboratory Diagnostics, 12th 5-Year Project of Shandong Province, Weifang Medical University, Weifang, Shandong 261053, China; ^3^Department of Pathology, Affiliated Hospital of Weifang Medical University, Weifang, Shandong 261053, China

## Abstract

The aim of the present study was to investigate the effects of phosphorylatable nucleus localization signal linked nucleic kinase substrate short peptide (^p^NNS)-conjugated chitosan (^p^NNS-CS) mediated miR-140 and IGF-1 in both rabbit chondrocytes and cartilage defects model. ^p^NNS-CS was combined with pBudCE4.1-IGF-1, pBudCE4.1-miR-140, and negative control pBudCE4.1 to form pDNA/^p^NNS-CS complexes. Then these complexes were transfected into chondrocytes or injected intra-articularly into the knee joints. High levels of IGF-1 and miR-140 expression were detected both* in vitro* and* in vivo*. Compared with pBudCE4.1 group,* in vitro*, the transgenic groups significantly promoted chondrocyte proliferation, increased glycosaminoglycan (GAG) synthesis, and ACAN, COL2A1, and TIMP-1 levels, and reduced the levels of nitric oxide (NO), MMP-13, and ADAMTS-5.* In vivo*, the exogenous genes enhanced COL2A1, ACAN, and TIMP-1 expression in cartilage and reduced cartilage Mankin score and the contents of NO, IL-1*β*, TNF-*α*, and GAG contents in synovial fluid of rabbits, MMP-13, ADAMTS-5, COL1A2, and COL10A1 levels in cartilage. Double gene combination showed better results than single gene. This study indicate that ^p^NNS-CS is a better gene delivery vehicle in gene therapy for cartilage defects and that miR-140 combination IGF-1 transfection has better biologic effects on cartilage defects.

## 1. Introduction

Articular cartilage has limited self-repair ability [1]. Gene therapy is a good candidate for articular cartilage repair and has become a hot topic for research [1–5]. The choice of gene delivery vehicle is crucial to gene therapy, and many studies have been undertaken to develop efficient and safe gene delivery vehicles [2–7]. As a polycationic nonviral gene delivery vehicle, chitosan (CS) has been studied by many researchers. The effects of gene delivery of CS nanoparticles carrying therapeutic genes, microRNA (miRNA), or siRNA have been studied both* in vitro* and* in vivo* [6–12]. The transfection efficiency of CS is low under physiological conditions [9]. Many researchers, including us, have attempted to improve the transfection efficiency of CS through chemical modifications to its structure [6–13]. In our previous study, we have confirmed that ^p^NNS-CS improved the pDNA transfection efficiency in C2C12 myoblast cells [13]. So we proposed that ^p^NNS-CS can be used in the study of gene therapy for cartilage defects as a gene delivery vehicle. The structure of chondrocytes is different from that of C2C12 cell, and previously we only studied the transfection efficiency of ^p^NNS-CS* in vitro*. So there are many problems need to be verified, such as how is the transfection efficiency of ^p^NNS-CS in chondrocyte and* in vivo*, whether the intra-articular injection administration affects the stability of the pDNA/^p^NNS-CS complex, and whether ^p^NNS-CS is a reliable and efficient gene delivery vehicle in cartilage defects gene therapy. Therefore, the present study was designed to evaluate ^p^NNS-CS as gene delivery vehicle carrying exogenous genes into chondrocyte both* in vitro* and* in vivo*.

Articular cartilage regeneration involves many anabolic growth factors, multiple growth factors gene combination therapy may augment articular cartilage repair, and many studies had combined with multiple genes to treat cartilage defects [1–4, 14, 15]. Because of their chondroregenerative effects, many cytokines regulate normal cartilage metabolism, many genes coding for IGF-I [1, 3, 4, 8, 15, 16], transforming growth factor-*β* (TGF-*β*) [2–4, 14, 15], bone morphogenetic protein-2 (BMP-2) and BMP-7 [3], transcription factor SOX9 [1, 2, 4], basic fibroblast growth factor (bFGF) [3, 16], and interleukin-1 receptor antagonist protein (IL-1Ra)[8, 14, 16] have been transferred into chondrocyte, and results have shown that the effects of combined gene transfer are superior to single gene [1–4, 8, 14–16]. IGF-1 is a mitogenic and anabolic factor that induces specific anabolic effects on maintaining cartilage metabolism and a stable environment, such as stimulating GAG, ACAN, and COL2A1 synthesis, stimulating chondrocyte proliferation, reducing chondrocyte catabolic activity, and maintaining the chondrocyte phenotype [1, 4, 8, 15–17]. miRNAs are small nonencoding genes [18]. Many studies have demonstrated miRNA dysregulation in osteoarthritis [19–21]. Many miRNAs play crucial functions in cartilage functional repairs [18, 22–26], especially the cartilage-specific miR-140. miR-140 affects many genes expression that regulate the extracellular matrix of cartilage, such as MMP13, TIMP1, ADAMTS-5, ACAN, COL2A1, COL1A2, and COL10A1. miR-140 can also promote the proliferation of chondrocytes and protect the injured cartilage cells [18, 25, 26]. These results show that IGF-1 and miR-140 can be selected for gene therapy in cartilage defects and combination of IGF-1 and miR-140 may achieve better therapeutic efficacy.

The goals of this study were to determine whether ^p^NNS-CS can carry IGF-1 and miR-140 genes into chondrocyte and efficient expression and the effects of exogenous genes both in cultured rabbit chondrocyte and in cartilage defects. The efficacies of the combination of IGF-1 and miR-140 have also been detected.

## 2. Materials and Methods

### 2.1. Reagents and Animals

CS was purchased from Sigma (MO, USA). Annexin V-FITC apoptosis detection kit, human IGF-1 ELISA kit, and DMEM/F12 medium were purchased from Thermo Fisher (Shanghai, China). MTT, PMSF, and RIPA we purchased from Solarbio Life Sciences (Beijing, China). IL-1*β* was purchased from PeproTech (NJ, USA). We also purchased the following kits: nitrate reductase kit of NO from NanJing JianCheng Bioengineering Institute (Nanjing, China); rabbit GAG ELISA kit from Nanjing Sen Beijia biotechnology Co., Ltd. (Nanjing, China); rabbit IL-1*β* and TNF-*α* ELISA kits from Shanghai MLBIO biotechnology Co., Ltd. (Shanghai, China); RNAiso Plus, SYBR Premix Ex Taq II, PrimeScript™ RT reagent Kit and Mir-X™ miRNA First-Strand Synthesis Kit from Takara (Dalian, China); and KOD-Plus-Ver polymerase from TOYOBO (Tokyo, Japan); ACAN, COL2A1, tissue inhibitor of metalloproteinases-1 (TIMP1), matrix metallopeptidase-13 (MMP-13), and a disintegrin and metalloproteinase with thrombospondin motifs 5(ADAMTS-5) antibodies from Bioss (Beijing, China). One-week-old and three-month-old New Zealand white rabbits (2.0-2.5 kg) were purchased from Jinan Jinfeng Experimental Animal Limited by Share Ltd. (Shandong, China).

### 2.2. IGF-1 and pri-miR-140 Plasmid Vectors

pBudCE4.1-IGF-1 containing hIGF-1 cDNA was previously constructed [8] and briefly described as follows: the coding regions of human IGF-I were amplified with PCR and directionally inserted into the* Xho*I and* Kpn*I sites of pBudCE4.1 plasmid to construct the expression plasmids pBudCE4.1-IGF-I. The sequence and genomic position of human mature miR-140 (miR-140) and pre-miR-140 were searched from the NCBI (https://www.ncbi.nlm.nih.gov/gene/) and Ensembl (http://asia.ensembl.org/index.html) and then flanked at both ends to obtain about 210 bp sequence of pri-microRNA-140 (pri-miR-140). Then the pri-miR-140 was amplified from human genomic DNA and subcloned into pBudCE4.1 to construct the expression plasmids pBudCE4.1-miR-140. A random sequence was subcloned into pBudCE4.1 plasmid serves as a negative control (pBudCE4.1). The pBudCE4.1-IGF-1, pBudCE4.1-miR-140, and pBudCE4.1 plasmids were purified with plasmid Kit (TIANGEN, Beijing, China).

### 2.3. Preparation of pDNA/^*p*^*NNS*-CS Complexes

The NNS (“PKKRKVREEAIKFSEEQRFRR”) contained a potentially phosphorylatable serine residue and a SV40 nucleus localization signal. This phosphorylatable NNS (^p^NNS) was conjugated to chitosan to form ^p^NNS-CS as previously described [13]. The plasmids of pBudCE4.1, pBudCE4.1-IGF-1, and pBudCE4.1-miR-140 were mixed, respectively, with ^p^NNS-CS in weight ratios of 1:0.5; 1:0.75; 1:1; 1:1.25; 1:1.5; 1:2; 1:2.5 to form the pDNA/^p^NNS-CS (pBudCE4.1/^p^NNS-CS, pBudCE4.1-IGF-1/^p^NNS-CS, and pBudCE4.1-miR-140/^p^NNS-CS) complexes as previously described [8]. Agarose gel electrophoresis assesses the pDNA/^p^NNS-CS complexes. In the subsequent study, the pDNA/^p^NNS-CS complexes were prepared at a 1:2 weight ratio of pDNA: ^p^NNS-CS. pEGFP-C1 plasmid was also mixed with CS or ^p^NNS-CS to form the pEGFP/CS and pEGFP/^p^NNS-CS complex. The transfection efficiency was evaluated by observing GFP-positive cells under fluorescence microscope.

### 2.4. *In Vitro* Experiment

#### 2.4.1. Isolation and Culture Transfection of Articular Chondrocytes

Articular chondrocytes were isolated from knees of both hind limbs of one-week-old rabbits and cultured as described previously [8], and in the following experiments, the second-generation chondrocytes were used. Chondrocytes were seeded on 96-well and 6-well microplates in complete DMEM/F12 containing 10% FBS in an incubator containing 5% CO2 at 37°C. Chondrocytes were treated with pDNA/^p^NNS-CS complexes when grown to 75% confluence. Chondrocytes were treated with IL-1*β* (10ng/mL) 24h after transfection.

The chondrocytes were divided into four groups: (1) pBudCE4.1/^p^NNS-CS transfected chondrocytes as negative control group (pBudCE4.1), (2) pBudCE4.1-IGF-1/^p^NNS-CS transfected chondrocytes (pBudCE4.1-IGF-1), (3) pBudCE4.1-miR-140/^p^NNS-CS transfected chondrocytes (pBudCE4.1-miR-140), and (4) pBudCE4.1-IGF-1/^p^NNS-CS combined pBudCE4.1-miR-140/^p^NNS-CS transfected chondrocytes (pBudCE4.1-IGF-1+miR-140). In the following experiments, the chondrocytes in the 6-well plates were used to detect the apoptosis of chondrocytes; the expression of exogenous mature miR-140 (m-miR-140); the expression of ACAN, COL2A1, TIMP-1, MMP-13, and ADAMTS-5; and the levels of NO, GAG, and exogenous IGF-1 in cell supernatant.

#### 2.4.2. Proliferation and Apoptosis of Chondrocytes

Chondrocytes proliferation was detected using a standard MTT method. Cells were seeded in 96-well microplates and transfected with pDNA/^p^NNS-CS complexes. MTT solution (15*μ*L; 5 mg/mL) was applied to each well after 48 hours transfection and incubated at 37°C for another 4 hours. Then dimethylsulfoxide (150 *μ*L/well) was added to each well, and the optical density at 570 nm was detected, and background optical density at 630 nm was subtracted. Each group experiment was repeated six times.

Chondrocytes apoptosis was detected using Annexin V-FITC labeling by flow cytometry (BD, USA) as described previously [8].

#### 2.4.3. NO, GAG, and IGF-1 Levels in Cell Supernatants

Cell supernatants were collected after 96-hour transfection and detected to determine the accumulation of NO by a nitrate reductase kit, the concentrations of GAG, and IGF-1 by ELISA kits according to the manufacturer's directions.

#### 2.4.4. Quantitative Real Time-PCR (qRT-PCR) Analysis

After 96 h transfection, cell were collected and used to extract total RNA using RNAiso Plus. Total RNA (2 *μ*g) was, respectively, reverse transcribed into total cDNA using Mir-X™ miRNA First-Strand Synthesis kit for detecting miR-140, and PrimeScript™ RT reagent Kit for detecting COL2A1, ACAN, TIMP-1, MMP-13, and ADAMTS-5 mRNA. The expression levels of m-miR-140, COL2A1, ACAN, TIMP-1, MMP-13, and ADAMTS-5 were detected via qRT-PCR in iQ5TM (BIO-RAD, USA). The primers are listed as follows: miR-140 (Forward: 5′-CGCGCCAGTGGTTTTACCCT-′3; the reverse primer and U6 reference gene primers were from reverse transcription kit), COL2A1 (Forward: 5′-ATGGCGGCTTCCACTTCAG-′3; Reverse: 5′-CGGTGGCTTCATCCAGGTAG -′3), ACAN (Forward: 5′-AGAACAGCGCCATCATTGC-′3; Reverse: 5′- CTCACGCCAGGGAACTCATC-′3), TIMP-1 (Forward: 5′-ATGGAAAGTGTCTGCGGGTAC-′3; Reverse: 5′-AGCCGGAACGTTGAGAGAAG-′3), MMP-13 (Forward: 5′-TGATGATGATGAAACTTGG-′3; Reverse: 5′-CATCAGGAAGCATAAAGTG-′3), ADAMTS-5 (Forward: 5′-TGTCCAATTTCGTGAGCC-′3; Reverse: 5′- TGTTCACCAGAGAGGATTTATG-′3), *β*-2-microglobulin (B2M) [27] (Forward: 5′-AACGTGGAACAGTCAGACC-′3; Reverse: 5′-AGTAATCTCGATCCCATTTC -′3). The raw CT values of m-miR-140 were calibrated to that of U6 reference gene and the CT values of the other genes were calibrated to the B2M; Delta-Delta Ct (∆∆CT) method was applied to calculate the genes expression.

#### 2.4.5. Western Blot Analysis

To detect the expression of collagen II, aggrecan, TIMP-1, MMP-13, and ADAMTS-5, chondrocytes transfection 96 h was lysed in RIPA lysis buffer (containing 0.1% PMSF). The concentration of protein was detected by bicinchoninic acid protein assay kit. The lysates were run on 8% SDS-polymerized gel and electrotransferred to PVDF membranes. The membranes were blocked in TBS-T containing 5% skimmed milk and incubated with primary antibody against collagen II (1:200), aggrecan (1:200), TIMP-1 (1:200), MMP-13 (1:200), ADAMTS-5 (1:200), and GAPDH (1:500, using as the loading control), according to standard immunoblotting protocols. Proteins were detected using enhanced chemiluminescence western blot detection kit (Millipore, Darmstadt, Germany) according to the manufacturer's guide and pictures were captured using the ChemiDocTM XRS+system (Bio-Rad, USA).

### 2.5. *In Vivo *Experiment

#### 2.5.1. Animals and Experimental Articular Cartilage Defect

Twenty-four three-month-old rabbits were randomly divided into four groups of six rabbits each. All four groups were made artificial cartilage full-thickness defects (4 mm diameter; 3 mm deep) as previously described [27] and received pDNA/pNNS-CS complexes. Following surgery, disinfection of the skin wounds and intramuscular injection of penicillin (400,000 U) were performed for 5 days. All rabbits were raised in separate cages under normal conditions, and allowed to exercise freely. Within 1 week after the operation, the joint activities of all rabbits in each group almost returned to normal. All procedures involving animals were approved by the Animal Care and Use Committee of China.

On the seventh day after surgery, isotonic saline and pDNA/^p^NNS-CS complexes dissolved in saline to adjust the volume to 0.2 mL were injected into rabbit joint cavities, and the injection groups were as follows: (i) group 1, negative control group (pBudCE4.1); (ii) group 2 (pBudCE4.1-IGF-1); (iii) group 3 (pBudCE4.1-miR-140); (iv) group 4 (pBudCE4.1-IGF-1+miR-140). The amount of pDNA in each group was 15 *μ*g (each time). The injection scheme was performed twice a week for 7 weeks.

Eight weeks after surgery, all experimental rabbits were again anaesthetized and 1mL isotonic saline was injected to lavage the joint space. The joint cavity lavage fluid (synovial fluid) was used to detect the levels of NO, GAG, and IGF-1. Then all rabbits were killed, dissected, and photographed. The area of defect and its surrounding cartilage tissue were collected and divided into two parts. One part was used to extract total RNA for qRT-PCR (n=6), and another part was used to histological evaluation (n=6).

#### 2.5.2. NO, IL-1*β*, TNF-*α*, GAG, and IGF-1 Levels in Synovial Fluids

The levels of NO, IL-1*β*, TNF-*α*, GAG, and exogenous IGF-1 in synovial fluid were detected as described above* in vitro* study.

#### 2.5.3. qRT-PCR Analysis of m-miR-140, COL2A1, COL1A2, COL10A1, ACAN, TIMP-1, MMP-13, and ADAMTS-5

Total RNA was extracted and reverse transcribed into cDNA, and the expression levels of m-miR-140, COL2A1, COL1A2, COL10A1, ACAN, TIMP-1, MMP-13, and ADAMTS-5 were detected via qRT-PCR as described above* in vitro* study. Primers of COL10A1 and COL1A2 had the following sequences: COL1A2 (Forward: 5′- GTGCTAAAGGAGAGAAAGGAAC -′3; Reverse: 5′- ACCAGGGAAACCAGTCATAC -′3), COL10A1 (Forward: 5′-CCCAGAACCCAGAATCCATC-′3; Reverse: 5′- ACTGTGTCTTGGTGTTGGGTTG-′3).

#### 2.5.4. Histological Assay of the Articular Cartilage

Following dissection, one-half of the cartilage defective area from each group was placed in the bottles containing 10% buffered formalin. After 24 h, the specimens were decalcified using 10% EDTA solution and embedded in paraffin. Serial sagittal sections were cut and stained with toluidine blue, Safranin O/fast green, and immunohistochemistry, following the standard operating procedure. Articular cartilage structure was observed using optical microscope (Olympus, Japan), and the severity of cartilage damage was graded histologically according to the Mankin scale [28].

### 2.6. Statistical Analysis

All results are reported as mean ± standard deviation (SD). Statistical significance was evaluated using SPSS 17.0 software. Multigroup comparisons were evaluated using the single-factor analysis of variance. Each result was compared using the Student-Newman-Keuls test.* P *<0.05 were considered to be statistically significant.

## 3. Results

### 3.1. Agarose Gel Electrophoresis and Transfection Efficiency of pDNA/^*p*^*NNS*-CS Complexes

**Figure 1:**
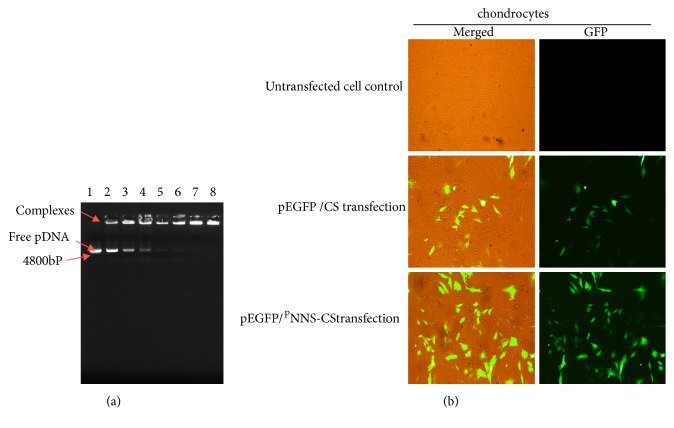
Electrophoresis and transfection efficiency of pDNA/^p^NNS-CS complex. (a) Detection of pDNA/^p^NNS-CS complex using agarose gel electrophoresis. Lane 1: free plasmid DNA; lanes 2-8: pDNA/^p^NNS^p^NNS-CS (w/w) = 1:0.5; 1:0.75; 1:1; 1:1.25; 1:1.5; 1:2; 1:2.5. When the ratio reached 1:2, pDNA/^p^NNS-CS lost mobility in the agarose gel. (b) GFP investigation at 72 h after transfection. After 72h of pEGFP/CS and pEGFP/^p^NNS-CS treated chondrocytes, GFP was detected using fluorescent microscope (×100).

When the ratio of pDNA:  ^p^NNS-CS was at or beyond 1:2, the pDNA/^p^NNS-CS complexes lost their mobility in the gel (Figure 1(a)). Fluorescent microscope showed that pEGFP-C1 was transfected into chondrocytes, and the ^p^NNS conjugation increases the expression of EGFP gene (Figure 1(b)).

### 3.2. In Vitro Results

#### 3.2.1. Effects of IGF-1 and miR-140 on IL-1*β* Treated Chondrocyte Proliferation and Apoptosis

**Figure 2:**
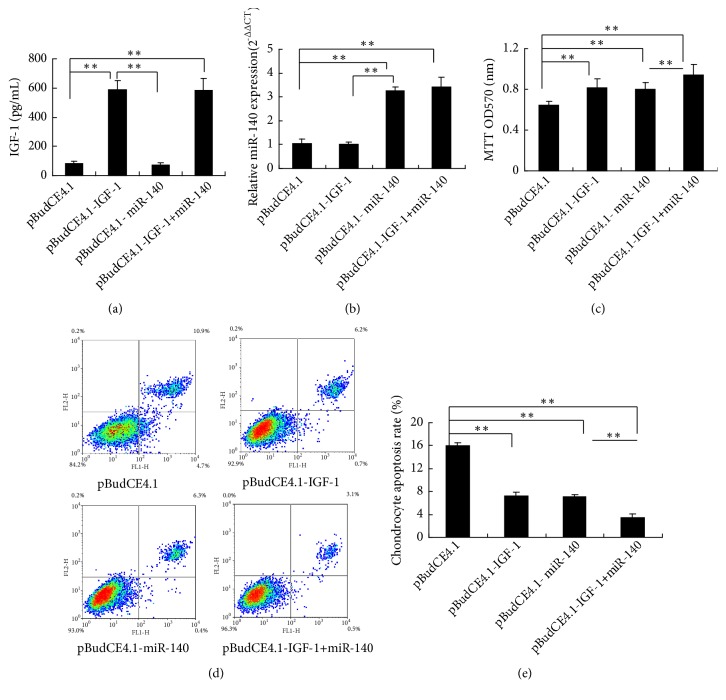
Analysis of expression IGF-1 and miR-140 on IL-1*β* treated rabbit chondrocytes proliferation and apoptosis. (a) IGF-1 produced in the cell supernatant. (b) The relative expression levels of miR-140 in four groups were examined using RT-qPCR. The gene expression of miR-140 for each group was normalized to the U6 expression levels. (c) MTT analyses of chondrocyte proliferation in vitro. (d-e) Annexin V-FITC analyses of chondrocyte apoptosis in vitro. Data are reported as the mean ± SD. ^*∗∗*^*p* < 0.01.

The expression levels of IGF-1 in the cell supernatants were similarly increased in the pBudCE4.1-IGF-1 and pBudCE4.1-IGF-1+miR-140 groups compared with the pBudCE4.1-miR-140 and pBudCE4.1 groups (*p *<0.05). There was no significant differences between the pBudCE4.1-miR-140 and pBudCE4.1 groups (*p *>0.05) (Figure 2(a)). The miR-140 expression levels in chondrocytes were similarly increased in the pBudCE4.1-miR-140 and pBudCE4.1-IGF-1+miR-140 groups compared with the pBudCE4.1-IGF-1 and pBudCE4.1 groups (*p *<0.05). There was no significant difference between the pBudCE4.1-IGF-1 and pBudCE4.1 groups (*p *>0.05) (Figure 2(b)). Compared with the pBudCE4.1 groups, chondrocyte proliferation was significantly increased in the pBudCE4.1-IGF-1+miR-140 group and then followed by the pBudCE4.1-IGF-1 group and pBudCE4.1-miR-140 group (*p *<0.05). There was no significant difference in promoting chondrocyte proliferation between the pBudCE4.1-miR-140 group and the pBudCE4.1-IGF-1 group (*p *>0.05) (Figure 2(c)). Compared with the pBudCE4.1 groups, chondrocyte apoptosis was significantly decreased in the pBudCE4.1-IGF-1+miR-140 group and then followed by the pBudCE4.1-IGF-1 group and pBudCE4.1-miR-140 group (*p *<0.05). There was no significant difference in inhibiting chondrocyte apoptosis between the pBudCE4.1-miR-140 group and the pBudCE4.1-IGF-1 group (*p *>0.05) (Figures 2(d) and 2(e)).

#### 3.2.2. NO and GAG Concentrations in Cell Supernatants

**Figure 3:**
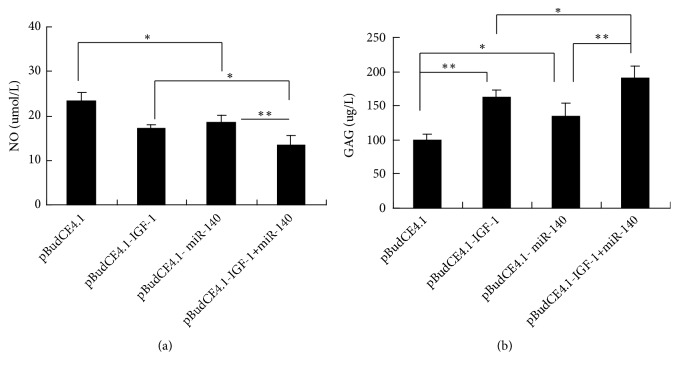
NO and GAG production in culture by IL-1*β* treated chondrocytes. (a) The NO levels in cell supernatant were measured by a nitrate reductase assay. (b) The GAG concentration in the cell supernatant was measured by ELISA. The data are shown as the means ± SD. ^*∗*^*p* < 0.05; ^*∗∗*^*p* < 0.01.

As shown in Figure 3(a), compared with the pBudCE4.1 group, the levels of NO were lowest in pBudCE4.1-IGF-1+miR-140 group and then followed by pBudCE4.1-IGF-1 group and pBudCE4.1-miR-140 group (*p *<0.05) (Figure 3(a)). The accumulation of GAG in the pBudCE4.1-IGF-1+miR-140 group was higher than the other three groups. Significantly more GAG also accumulated in the pBudCE4.1-IGF-1 group and pBudCE4.1-miR-140 group supernatants than in the pBudCE4.1 group supernatants (*p *<0.05) (Figure 3(b)). There was no significant difference of GAG and NO levels between the pBudCE4.1-IGF-1 group and pBudCE4.1-miR-140 group (*p *>0.05) (Figures 3(a) and 3(b)).

#### 3.2.3. qRT-PCR and Western Blot Quantitative ACAN, COL2A1, TIMP-1, MMP-13, and ADAMTS-5 Expression in IL-1*β* Treated Chondrocytes

**Figure 4:**
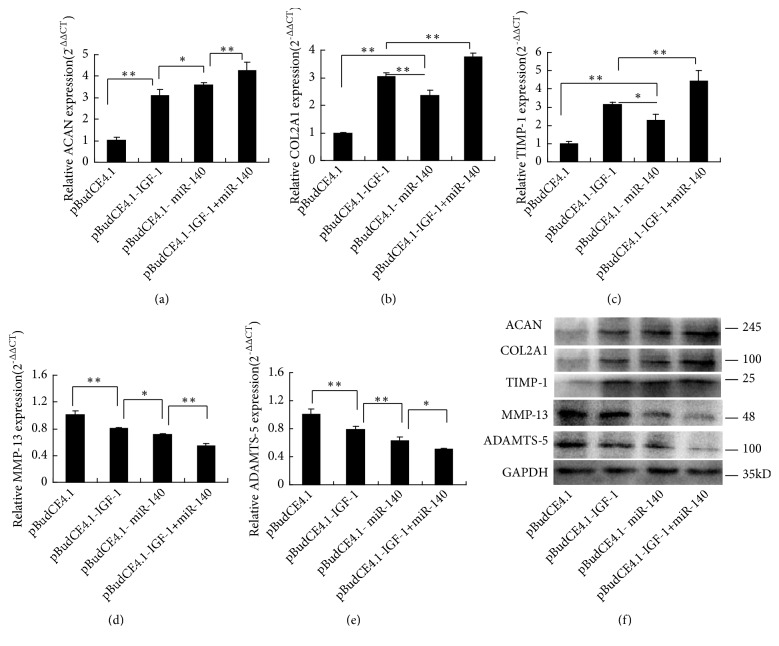
The expression of ACAN, COL2A1, TIMP-1, MMP-13, and ADAMTS-5 in IL-1*β* treated chondrocytes. (a-e) RT-qPCR assays of the mRNA expression of ACAN, COL2A1, TIMP-1, MMP-13, and ADAMTS-5. The expression data of raw mRNA genes for each group were normalized to the B2M expressions levels, and the relative expression level of each gene is represented as 2^−∆∆CT^. The data are reported as the means ± SD. ^*∗*^*p* < 0.05; ^*∗∗*^*p* < 0.01. (f) Western blot assays protein expression of ACAN, COL2A1, TIMP-1, MMP-13, and ADAMTS-5. GAPDH was used as the internal control.

Compared with the pBudCE4.1 group, ACAN, COL2A1, and TIMP-1 expressions were significantly increased and MMP-13 and ADAMTS-5 expressions were decreased statistically in the pBudCE4.1-IGF-1+miR-140 group and then followed by the pBudCE4.1-IGF-1 group and pBudCE4.1-miR-140 group (*p*<0.05). Moreover, compared with the pBudCE4.1-miR-140 group, COL2A1, TIMP-1, ADAMTS-5, and MMP-13 expressions were significantly higher and ACAN was lower in the pBudCE4.1-IGF-1 group (*p *<0.05) (Figures 4(a)–4(e)). To further confirm the effect of both IGF-1 and miR-140 on chondrocytes, Western blot was used to determine the expression level of ACAN, COL2A1, TIMP-1, MMP-13, and ADAMTS-5 protein. Western blot results show that the protein expression levels of these genes (Figure 4(f)) are consistent with their mRNAs findings.

### 3.3. In Vivo Results

#### 3.3.1. IGF-1, NO, GAG, IL-1*β*, and TNF-*α* Concentrations in the Synovial Fluids

**Figure 5:**
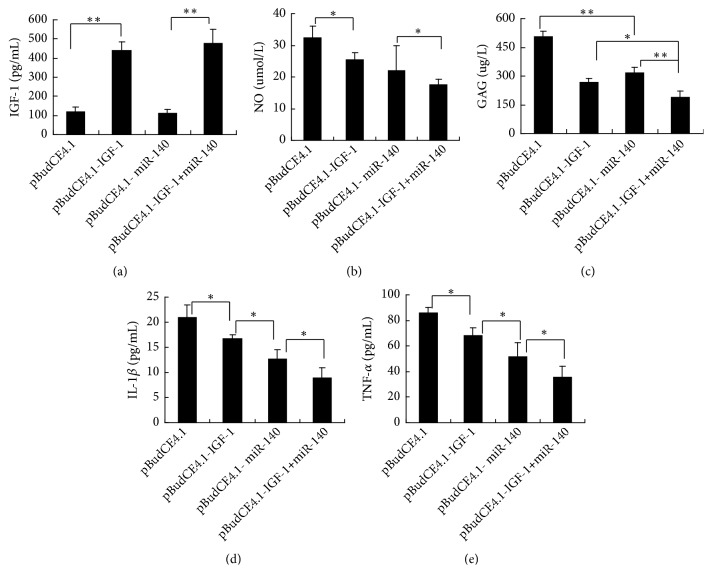
Concentrations of IGF-1, NO, GAG, IL-1*β*, and TNF-*α* in synovial fluids. (a) The ELISA results of exogenous IGF-1 concentrations in synovial fluids. (b) The nitrate reductase assay results of NO concentrations in synovial fluid. (c-e) The ELISA results of GAG, IL-1*β*, and TNF-*α* concentrations in synovial fluid. The data are reported as the means ± SD. ^*∗*^*p* < 0.05; ^*∗∗*^*p* < 0.01.

The pBudCE4.1-IGF-1 group and the pBudCE4.1-IGF-1+miR-140 group showed significantly similar higher IGF-1 concentrations than the pBudCE4.1 group (*p* <0.05); There was no significant difference of IGF-1 concentrations between the pBudCE4.1-miR-140 group and pBudCE4.1 group (*p *>0.05) (Figure 5(a)). Compared with pBudCE4.1 group, all of the transgenic groups showed reduced NO, GAG, IL-1*β*, and TNF-*α* concentrations. The NO, GAG, IL-1*β*, and TNF-*α* contents in the synovial fluids of the pBudCE4.1-IGF-1+miR-140 group were the lowest in all groups (*p* <0.05) (Figures 5(b)–5(e)). There was no significant difference of NO and GAG levels between the pBudCE4.1-IGF-1 group and pBudCE4.1-miR-140 group (*p *>0.05) (Figures 5(b) and 5(c)). In the pBudCE4.1-miR-140 group, the IL-1*β* and TNF-*α* contents in the synovial fluids were statistically lower than in the pBudCE4.1-IGF-1 group (*p* <0.05) (Figures 5(d) and 5(e)).

#### 3.3.2. Quantitative miR-140, ACAN, COL2A1, COL1A2, COL10A1, TIMP-1, MMP-13, and ADAMTS-5 Expression in Cartilage

**Figure 6:**
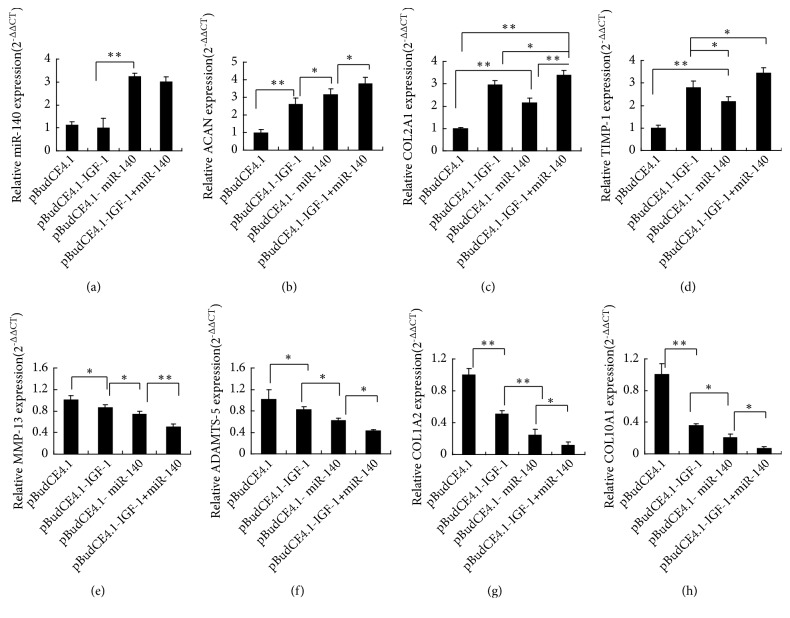
Cartilage expression of miR-140, ACAN, COL2A1, COL1A2, COL10A1, TIMP-1, MMP-13, and ADAMTS-5. RT-qPCR assays of the expression of miR-140, ACAN, COL2A1, COL1A2, COL10A1, TIMP-1, MMP-13, and ADAMTS-5. (a) The raw expression data of miR-140 for four groups were calibrated to the U6. (b-h) The raw expression data of ACAN, COL2A1, COL1A2, COL10A1, TIMP-1, MMP-13, and ADAMTS-5 for four groups were calibrated to the B2M. ^*∗*^*p* < 0.05; ^*∗∗*^*p* < 0.01.

Compared with the other groups, the expression levels of miR-140 in cartilage were similarly higher in pBudCE4.1-IGF-1+miR-140 group and pBudCE4.1-miR-140 group (*p* <0.05). No significant difference was detected between the pBudCE4.1 group and pBudCE4.1-IGF-1 group (*p* >0.05) (Figure 6(a)). Compared with pBudCE4.1 group, the expressions of ACAN, COL2A1, and TIMP-1 were significantly up-regulated, and expressions of MMP-13, ADAMTS-5, COL1A2, and COL10A1 were significantly downregulated in transgenic groups (*p* <0.05), and pBudCE4.1-IGF-1+miR-140 group has the strongest effect (*p* <0.05) (Figures 6(b)–6(h)). Moreover, in the pBudCE4.1-IGF-1 group, the expression of COL2A1, COL1A2, COL10A1, TIMP-1, MMP-13, and ADAMTS-5 mRNA were statistically higher and ACAN expression was significantly lower than in the pBudCE4.1-miR-140 group (*p* <0.05) (Figures 6(b)–6(h)).

#### 3.3.3. Gross Observation and Histologic Analysis of Articular Cartilage

**Figure 7:**
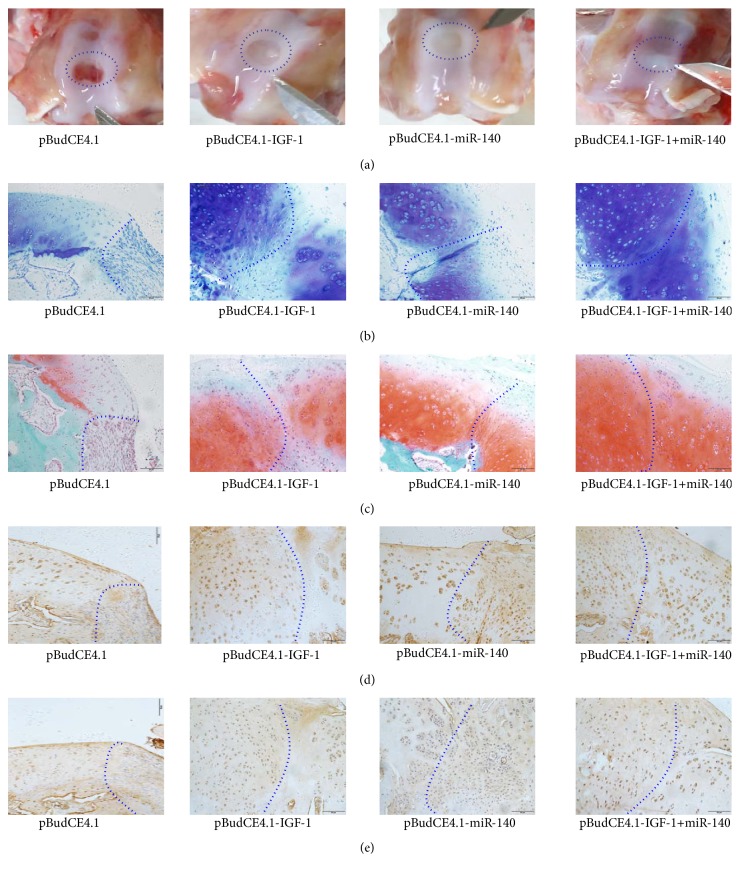
Gross observation and histologic analysis of articular cartilage change in rabbits. (a) Gross appearance of articular cartilage defects in the knees of the rabbit. (b) Sagittal section of femur trochlea was stained with toluidine blue. (c) Sagittal sections of femur trochlea were stained with Safranin O/fast green. (d and e) Immunohistochemical detection of ACAN and COL2A1 (×200). Dash lines highlight the actual border of articular cartilage defects.

*Gross Observation*. In the pBudCE4.1 group, there was almost no obvious cartilage-like tissue filling in the defects. The defects in the pBudCE4.1-IGF-1, pBudCE4.1-miR-140, and pBudCE4.1-IGF-1+miR-140 groups were covered with different degrees of white cartilage-like tissue. Especially in pBudCE4.1-IGF-1+miR-140 groups, the neocartilage was smooth, shiny, and boundary blurred with the surrounding normal cartilage tissue (Figure 7(a)).* Toluidine Blue Staining*. In the pBudCE4.1 group, fibrous tissue and inflammatory cells partially filled the defect; different degrees of cartilage-like tissue appeared in the pBudCE4.1-miR-140, pBudCE4.1-IGF-1, and pBudCE4.1-IGF-1+miR-140 groups. The most complete repair appeared in pBudCE4.1-IGF-1+miR-140 group, which were almost completely filled with nascent cartilage, and the articular cartilage surface was relatively continuous and smooth, the extracellular matrix (ECM) was almost uniformly stained, and the laminar structure was relatively clear (Figure 7(b)).* Safranin O/Fast Green Staining*. Compared with negative Safranin O staining in the defects area of pBudCE4.1 group, different intensities of Safranin O staining were detected in the defects area of the pBudCE4.1-IGF-1, pBudCE4.1-miR-140, and pBudCE4.1-IGF-1+miR-140 groups (Figure 7(c)). Nearly normal uniform Safranin O/fast green staining and structural organization appear in the pBudCE4.1-IGF-1+miR-140 group. Mankin scores were listed in Table 1.* Immunohistochemistry*. In the pBudCE4.1 group defect area, fibrous tissue and inflammatory cells generated a slight, nonspecific staining. In the pBudCE4.1-IGF-1, pBudCE4.1-miR-140, and pBudCE4.1-IGF-1+miR-140 groups defect areas, a large amount of ACAN and COL2A1 staining dark brown was detected in the new cartilage ECM, and the chondrocyte staining and morphology in the defects area of pBudCE4.1-IGF-1+miR-140 group are close to that of the normal surrounding chondrocytes (Figures 7(d) and 7(e)).

## 4. Discussion

CS can cross-link with collagen macromolecules [29]. Chondrocytes and collagen are exposed when cartilage is damaged, which facilitates the CS nanoparticles localization and exogenous genes expression in the defect, resulting in a therapeutic effect [30]. Our previous studies use ^p^NNS (contained a potentially phosphorylatable serine residue and a SV40 nucleus localization signal) conjugated chitosan (^p^NNS-CS) as gene delivery vehicle and in* in vitro *experiments have confirmed that ^p^NNS-CS could carry more pDNA into the nucleus of C2C12 cells and enhance exogenous genes expression, which is mainly related to the fact that ^p^NNS can promote exogene nuclear localization and intranucleus disassociation [13]. The results drive us to use ^p^NNS-CS as gene delivery vehicle in chondrocytes. Thus, the goal of this study is to further survey the effects of using ^p^NNS-CS mediated gene transfection in chondrocyte, as well as the effects on cartilage defects repair. In this study, we first verified that ^p^NNS-CS can improve transfection efficiency in chondrocytes. Second the results demonstrate that ^p^NNS-CS mediated pBudCE4.1-IGF-1, pBudCE4.1-miR-140, or pBudCE4.1-IGF-1+miR-140 genes transfection in chondrocytes can induce IGF-1 and miR-140 overexpression both* in vitro *and* in vivo*.

Since IGF-1 promotes chondrocyte proliferation and ECM synthesis, it has been widely used in cartilage defect repair and has made encouraging results [4, 8, 15, 16]. Many miRNAs play critical roles in cartilage-specific processes [31–33]. miR-140 inhibits the degradation of cartilage ECM by inhibiting ADAMTS5 and MMP13 expression [18, 25, 34, 35]. Osteoarthritis and inflammatory signaling associated with cartilage degradation reduce miR-140 expression [34, 36]. miR-140^−/−^ mice show early osteoarthritic changes onset in various cartilages, and miR-140 transgenic mice are resistant to arthritis induction [35, 37]. Many studies have attempted to use miR-140 to interfere with cartilage-related diseases [18, 25, 26, 37, 38]. In view of the roles of IGF-1 and miR-140 in chondrocytes, we proposed that IGF-1 and miR-140 may have hopeful potential as therapeutic targets for cartilage defects treatment. Our experimental results show that introduction of IGF-1 and miR-140 by ^p^NNS-CS transfection has a positive synergistic effect both* in vitro *and* in vivo*, and these effects of combination of two genes are obviously better than that of single gene.

NO as an inflammatory mediator and catabolizing factor is closely related to the damage of cartilage, which can inhibit proteoglycan and COL2A1 synthesis and induce matrix metalloproteinase (MMP_S_) synthesis in chondrocytes. High NO concentrations significantly induce chondrocyte apoptosis and decrease chondrocyte vitality [39, 40], so inhibition of NO production is a potential strategy for the treatment of cartilage damage. In this study, the outcomes showed that overexpression of IGF-1 and miR-140 inhibits NO production, inhibits apoptosis, and promoted chondrocyte against of IL-1*β* antiproliferative effect, and IGF-1 and miR-140 jointly significantly enhance these effects both* in vitro* and* in vivo*. TNF-*α* and IL-1*β* have been demonstrated to be important for cartilage degeneration. In this study, the outcomes showed that overexpression of IGF-1 and miR-140 reduces the content of TNF-*α* and IL-1*β* in the synovial fluid, and IGF-1 and miR-140 jointly significantly enhance these effects* in vivo*.

ACAN, COL2A1, and GAG are known to be the most components of cartilage ECM.* In vitro,* ACAN, COL2A1, and GAG biosynthesis support chondrocyte redifferentiation [41]. Therefore, changes in ACAN, COL2A1, and GAG reflect the anabolism of cartilage ECM. In this study,* in vitro*, overexpression of IGF-1 and miR-140 each individually promoted GAG accumulation in the cell supernatant and chondrocyte expression of ACAN and COL2A1, and these effects were significantly enhanced in IGF-1 and miR-140 jointly group.* In vivo,* cartilage damage causes GAG to release into synovial fluids. So, the changes of GAG levels in synovial fluids reflect catabolic activity in cartilage ECM [42]. In this study, overexpression of IGF-1 and miR-140 each individually reduces the content of GAG in synovial fluid, decreases COL1A2 (fibrocartilaginous markers) and COL10A1 (cartilage hypertrophy markers) synthesis [43, 44], and increases ACAN and COL2A1 synthesis compared with the negative control group (pBudCE4.1 transfection group) which were beneficial for cartilage repair, and fewer GAG in the synovial fluid, less COL1A2 and COL10A1, and more ACAN and COL2A1 synthesis were detected in IGF-1 combined with miR-140 group. These results imply that the repair tissue filling in the cartilage defects possesses characteristics of hyaline cartilage, and IGF-1 and miR-140 have synergistic effects for better therapeutic efficacy.

ECM degrading enzymes, such as the matrix metalloproteinase-13 (MMP-13) and a metalloproteinase with thrombospondin Motifs-5 (ADAMTS-5), play important roles in cleaving ACAN and COL2A1 [6, 45] and were involved in progressive erosion of articular cartilage. TIMP-1 is an inhibitor of MMPs activity during articular cartilage degeneration [46], which promotes cell proliferation and reduces cell apoptosis [18]. IGF-1 [16, 45] and miR-140 [18, 26, 34, 35, 38] both can significantly reduce MMP-13 and ADAMTS-5 expression and increase TIMP-1 level, thus inhibiting the degeneration of ACAN and COL2A1 of ECM. In this study, IGF-1 and miR-140 each individually shows that beneficial effects on MMP-13, ADAMTS-5, and TIMP-1 are consistent with these results of previous studies [16, 18, 26, 34–38, 47], and more beneficial effects were detected in IGF-1 and miR-140 jointly group; these may explain their mediated ECM production and chondrocyte proliferation.

Histological analysis also showed that intra-articular gene delivery of IGF-1 and miR-140 significantly lowered the Mankin score of defect cartilage, promoted ACAN, COL2A1, and GAG synthesis in the ECM, and diminished COL1A2 and COL10A1 staining intensities in the newly formed cartilage tissue. All results strongly suggest that the synergistic functions in promoting functional recovery of IGF-1 and miR-140 double transfection were obviously better than either of the single transfections, not only for inhibiting the inflammatory response, cartilage degradation, chondrocyte hypertrophy, and fibrous cartilage formation but also for promoting cartilage proliferation.

## 5. Conclusions

Our study verified that ^p^NNS-CS can efficiently carry exogenous genes into chondrocytes and expression. Meanwhile, these study results provided the direct experimental evidences that gene therapy using IGF-1 and miR-140 was valid in repairing of cartilage defects, and combinations of IGF-1 and miR-140 have better biologic effects valid in improving repairing of articular cartilage and inhibiting degradation of articular cartilage. Our findings also provide a suitable experimental basis for articular cartilage defects gene therapy* in vivo* in the future.

## Figures and Tables

**Table 1:** A list of Mankin scores of cartilage specimens in groups (point).

Group	n	$\overset{-}{\chi }$±S
pBudCE4.1	6	10.33 ± 1.51^*a*^
pBudCE4.1-IGF-1	6	7.17 ± 0.75^*b*^
pBudCE4.1-miR-140	6	5.67 ± 1.212^*c*^
pBudCE4.1-IGF-1+miR-140	6	3.33 ± 1.03^*d*^

*a*, *b,c, d,* and *e *represent the Mankin scores from each group compared with *p* <0.05.

## Data Availability

The data used to support the findings of this study are included within the article.
